# Shear stress modulates macrophage-induced urokinase plasminogen activator expression in human chondrocytes

**DOI:** 10.1186/ar4215

**Published:** 2013-04-18

**Authors:** Chih-Chang Yeh, Shun-Fu Chang, Ting-Ying Huang, Hsin-I Chang, Hsing-Chun Kuo, Yi-Chien Wu, Ching-Hsiang Hsieh, Chung-Sheng Shi, Cheng-Nan Chen

**Affiliations:** 1 Graduate Institute of Clinical Medical Sciences, College of Medicine, Chang Gung University, Taoyuan 333, Taiwan; 2 Orthopaedic Department, Chiayi Branch, Taichung Veterans General Hospital, Chiayi 600, Taiwan; 3 Biophotonics & Molecular Imaging Research Center, National Yang Ming University, Taipei 112, Taiwan; 4 Department of Biochemical Science and Technology, National Chiayi University, Chiayi 600, Taiwan; 5 Institute of Nursing and Department of Nursing, Chang Gung University of Science and Technology; Chronic Diseases and Health Promotion Research Center, CGUST, Chiayi 613, Taiwan; 6 Orthopaedic Department, Yangming Hospital, Chiayi 600, Taiwan

## Abstract

**Introduction:**

Synovial macrophages, which can release proinflammatory factors, are responsible for the upregulation of cartilage-breakdown proteases and play critical roles in cartilage degradation during the progression of osteoarthritis (OA). In addition, shear stress exerts multifunctional effects on chondrocytes by inducing the synthesis of catabolic or anabolic genes. However, the interplay of macrophages, chondrocytes, and shear stress during the regulation of cartilage function remains poorly understood. We investigated the mechanisms underlying the modulation of human chondrocyte urokinase plasminogen activator (uPA) expression by macrophages and shear stress.

**Methods:**

Human chondrocytes were stimulated by peripheral blood-macrophage- conditioned medium (PB-MCM), or exposure of chondrocytes cultured in PB-MCM to different levels of shear stress (2 to 20 dyn/cm^2^). Real-time polymerase chain reaction was used to analyze uPA gene expression. Inhibitors and small interfering RNA were used to investigate the mechanism for the effects of PB-MCM and shear stress in chondrocytes.

**Results:**

Stimulation of human chondrocytes with PB-MCM was found to induce uPA expression. We demonstrated that activation of the JNK and Akt pathways and NF-κB are critical for PB-MCM-induced uPA expression. Blocking assays by using IL-1ra further demonstrated that IL-1β in PB-MCM is the major mediator of uPA expression in chondrocytes. PB-MCM-treated chondrocytes subjected to a lower level of shear stress showed inhibition of MCM-induced JNK and Akt phosphorylation, NF-κB activation, and uPA expression. The PB-MCM-induced uPA expression was suppressed by AMP-activated protein kinase (AMPK) agonist. The inhibitor or siRNA for AMPK abolished the shear-mediated inhibition of uPA expression.

**Conclusions:**

These data support the hypothesis that uPA upregulation stimulated by macrophages may play an active role in the onset of OA and in the shear-stress protection against this induction.

## Introduction

Osteoarthritis (OA) is the most common worldwide articular disease and affects a large number of adults. It results from articular cartilage failure induced by the interactions of genetic, metabolic, biochemical, and biomechanical factors with the secondary components of inflammation [1]. The processes underlying OA involve interactive degradation and repair systems in cartilage, bone, and the synovium. It is also now believed that synovial inflammation and the production of proinflammatory or destructive mediators from the OA synovium are important for the progression of OA [2]. Synovial tissues from patients with early signs of OA show infiltrations of macrophages that exhibit an activated phenotype and produce proinflammatory cytokines, mainly interleukin (IL)-1β and tumor necrosis factor (TNF)-α. Macrophage-derived IL-1β and TNF-α are required for the release of matrix metalloproteinases (MMPs) from the synovium that will ultimately degrade cartilage tissues [3]. It also has been observed that macrophages mediate osteophyte formation and fibrosis in the early stages of experimentally induced OA [4]. However, the effects of macrophages on human chondrocyte catabolic gene expression remain unclear.

Cartilage is a flexible connective tissue consisting of chondrocytes and an extracellular matrix (ECM). The cartilage-specific ECM is a dynamic and complex network consisting of water, collagen, and proteoglycan MMPs, and other small molecules, and it plays an essential role in cartilage structure and function [5]. In the processes that involve the proteolytic degradation of cartilage, the plasminogen activator (PA) system has been suggested as playing a key role in ECM remodeling [6]. This system is composed of urokinase-type PA (uPA), tissue-type PA (tPA), uPA receptor (uPAR), and PA inhibitor-1 (PAI-1). uPA is a 55-kDa serine protease, which is released as an inactive single-chain zymogen (pro-uPA). When bound to its receptor, uPAR, pro-uPA is activated and converts plasminogen into plasmin [7]. It has been reported that uPA can be upregulated in synovial fibroblasts from both OA and rheumatoid arthritis samples [8]. However, the molecular mechanisms underlying uPA expression in human chondrocytes remain unknown.

OA can result from mechanical injury to articular cartilage. Chondrocytes in cartilage tissue are constantly exposed to a variety of different mechanical forces that modulate gene expression and metabolic activity in these cells [9]. Previous studies have revealed that chondrocytes of the articular cartilage are exposed to different levels of fluid flow [10,11], suggesting that mechanical shear stress may be of pathophysiologic relevance in cartilage biology. In addition, the development of chondrocyte/cartilage tissue-engineering constructs is affected by different shear-stress ranges, revealing that fluid shear stress may alter the intercellular signaling pathways in chondrocytes [12,13]. Our previous study also indicated that shear stresses at 5 and 10 dyn/cm^2 ^(1 dyn = 10 μN) play an important role in the regulation of PAI-1 expression in human OA nonlesioned, but not lesioned, chondrocytes [14]. These data indicate that the nature and magnitude of shear stress may play a significant role in the homeostasis of the structure and function of cartilage.

The mechanical loading and inflammation in the joint that cause cartilage breakdown are believed to be important factors in the progression of OA. However, the mechanisms underlying macrophage-induced uPA expression in human chondrocytes, and the role of shear stress in the modulation of macrophage-induced gene expression, are still not understood. In our present study, we investigated the interplay between shear stress and inflammatory stimulation in modulating chondrocyte catabolic gene expression by analyzing the effects of shear stress on peripheral blood-macrophage-conditioned medium (PB-MCM)-induced uPA expression in human chondrocytes. In addition, PB-MCM-induced uPA expression was modulated by AMP-activated protein kinase (AMPK): an AMPK agonist suppressed PB-MCM-induced uPA expression, and inhibition of AMPK attenuated shear stress-inhibition of uPA expression. These findings concerning the mechanisms of suppression of PB-MCM-induced responses in chondrocytes by shear stress provide new insights into the pathophysiology of OA.

## Materials and methods

### Reagents

All culture materials were purchased from Gibco (Grand Island, NY, USA). PD98059 (ERK inhibitor), SP600125 (JNK inhibitor), SB203580 (p38 inhibitor), LY294002 (PI3K/Akt inhibitor), IL1ra (IL-1-receptor antagonist), tanshinone IIA (AP-1 inhibitor), 5-aminoimidazole-4-carboxamide 1-β-D-ribonucleoside (AICAR, the AMPK activator), and compound C (AMPK inhibitor) were purchased from Calbiochem (La Jolla, CA, USA). Mouse monoclonal antibodies (mABs) against JNK and phospho-JNK were purchased from Santa Cruz Biotechnology (Santa Cruz, CA, USA). Rabbit polyclonal antibodies against Akt and mAB against phospho-Akt were purchased from Cell Signaling Technology (Beverly, MA, USA). Neutralizing mABs against TNF-α were purchased from R&D Systems (Minneapolis, MN, USA). Human uPA enzyme-linked immunosorbent assay (ELISA) kits were obtained from American Diagnostica (Greenwich, CT, USA). ERK, JNK, p38, and AMPK siRNA vectors, and a control siRNA construct (containing random DNA sequences) were purchased from Invitrogen (Carlsbad, CA, USA). SN50 (NF-κB inhibitor) was obtained from Biomol Research Laboratories (Plymouth Meeting, PA, USA). All other chemicals of reagent grade were obtained from Sigma (St. Louis, MO, USA).

### Culture of human chondrocytes

Normal human chondrocytes were purchased from PromoCell (Heidelberg, Germany). Cells were grown in complete chondrocyte growth medium supplemented with 10% FBS. Cells at passage 2 or 3 were tested to ensure that they expressed collagen type II before use in the experiments. After reaching 80% confluency, the cells were trypsinized and seeded onto glass slides.

### Isolation of peripheral blood monocytes

Human monocytes from the buffy coat (Tainan Blood Center, TBSF, Taiwan) were isolated as previously described [15]. In brief, peripheral blood mononuclear cells (PBMCs) were isolated with Histopaque 1077 density-gradient centrifugation. Monocytes were then purified from PBMCs by negative selection by using a magnetic-activated cell sorting (MACS) monocyte isolation kit (Miltenyi Biotech, Auburn, CA, USA).

### Preparation of peripheral blood monocyte-derived macrophage-conditioned medium (PB-MCM)

Peripheral blood monocyte-derived macrophages were counted and plated at 5 × 10^5 ^cells/well on cell-culture dishes. For the collection of PB-MCMs for the culturing of peripheral blood monocyte-derived macrophages, freshly isolated peripheral blood monocytes were plated in 10% FBS. After 5 days in culture, the monocyte-derived macrophages were incubated for a further 48 hours in fresh serum-free RPMI medium. The conditioned media were then collected and defined as PB-MCM.

### Shear-stress experiment

Glass slides onto which cultured chondrocytes were mounted in a parallel-plate flow chamber were previously characterized and described in detail [16,17]. The chamber was connected to a perfusion loop system and maintained at 37°C in a temperature-controlled enclosure. The perfusate was maintained at pH 7.4 by continuous gassing with a humidified mixture of 5% CO_2 _in air. The fluid shear stress (τ) generated on the cells by flow was estimated to be 2 to 20 dyn/cm^2 ^by using the formula τ = 6 μ*Q/wh*^2^, where μ is the dynamic viscosity of the perfusate, *Q *is the flow rate, and *h *and *w *are the channel height and width, respectively.

### Real-time quantitative PCR

Total RNA preparations and RT reactions were carried out as described previously [14]. Gene expression was analyzed by quantitative real-time PCR (ABI Prism 7900HT) by using the SYBR Green PCR Master Mix (Applied Biosystems). The primers used were as follows: uPA forward, 5'-GCTCA CCACA ACGAC ATTGC-3'; uPA reverse, 5'-CACCT GCCCT CCTTG GAA-3'; and 18S rRNA forward, 5'-CGGCG ACGAC CCATT CGAAC-3', 18S rRNA reverse, 5'-GAATC GAACC CTGAT TCCCC GTC-3'. Values were normalized to the levels of 18S rRNA. All primer pairs had at least one primer that crossed an exon-exon boundary. Real-time PCR reactions were performed in triplicate and quantified by using the 2^−ΔΔCt ^method.

### Quantification of uPA expression

Release of uPA into culture media was analyzed by using commercially available ELISA kits purchased from American Diagnostica, Inc. The assays and data calculations were performed according to the manufacturer's instructions.

### Western blot analysis

Cells were lysed with a buffer containing 1% NP-40, 0.5% sodium deoxycholate, 0.1% SDS, and a protease inhibitor mixture (containing PMSF, aprotinin, and sodium orthovanadate). The total cell lysate (50 μg of protein) was separated with SDS-polyacrylamide gel electrophoresis (PAGE) (12% running, 4% stacking) and analyzed by using the designated antibodies and the Western-Light chemiluminescent detection system (Bio-Rad, Hercules, CA), as previously described [18].

### Reporter gene constructs, siRNA, transfection, and luciferase assays

The dominant-negative mutant of Akt (DN-Akt) was kindly provided by Dr. Yi-Shuan Li (University of California, San Diego). Human uPA promoter constructs containing the -2,350/+30, -1,872/+30, -1,700/+30, and -670/+30 5'-flanking regions of uPA were linked to the firefly luciferase reporter gene in the pGL4 vector (Promega, Madison, WI, USA), as previously reported [19]. uPA promoter fragments containing mutations in the NF-κB binding sites (GG to TT) were produced by site-directed mutagenesis. DNA plasmids at a concentration of 1 mg/ml were transfected into cells by using Lipofectamine (Gibco). The pSV-β-galactosidase plasmid was cotransfected to normalize for the transfection efficiency.

For siRNA transfection, cells were transfected with the designated construct by using a RNAiMAX transfection kit (Invitrogen). ERK-, JNK-, and p38-siRNA transfections caused at least an 80% reduction in the respective protein-expression levels compared with the siRNA control vector.

### Chromatin immunoprecipitation assay (ChIP)

ChIP assays were performed by using assay kits from Santa Cruz Biotechnology. Cells were fixed with 1% formaldehyde for 10 minutes, washed, and then harvested in SDS lysis buffer. After sonication, lysates containing soluble chromatin were immunoprecipitated by using 2 μg of antibodies against IgG or NF-κB p65. DNA was purified with a PCR Purification Kit (Qiagen) and used for PCR analysis. The amplified DNA fragments were visualized on an agarose gel. Primers that amplify regions of the human uPA promoter that contain the NF-κB binding sites (-1,859/-1,707) were used as follows: 5'-CCTGA TAACT AACCT GGGAG TTTC-3' and 5'-CTTCA GAGCC AACCT TGCTA CTTC-3'.

### Transcription factor ELISA assay

Nuclear extracts were prepared as previously described [19] and used for quantitative measurements of NF-κB p65 activation by using a commercially available ELISA kit (Panomics, Redwood City, CA, USA).

### Statistical analysis

The results shown in this study are expressed as mean ± standard error of the mean (SEM). Statistical analysis was performed by using an independent Student *t *test for two groups of data and analysis of variance (ANOVA) followed by the Scheffé test for multiple comparisons. *P *values of less than 0.05 were considered significant.

## Results

### Conditioned medium from macrophages induces the upregulation of uPA in human chondrocytes

**Figure 1:**
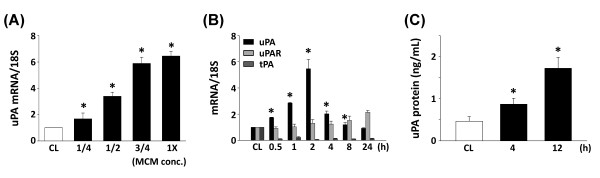
**Induction of urokinase-type PA (uPA) in chondrocytes by peripheral blood-macrophage-conditioned medium (PB-MCM) stimulation**. Chondrocytes were maintained as controls (CL) or stimulated with PB-MCM. Data are the mean ± SEM. *P < 0.05 versus CL chondrocytes. **(A) **The dose-dependent induction of uPA mRNA expression by PB-MCM. Human chondrocytes were grown as CL or were stimulated with different concentrations of PB-MCM for 2 hours. **(B) **RNA samples were isolated from human chondrocytes at the indicated times and subjected to real-time PCR analysis. The mRNA data are presented as the fold changes in fluorescent density from CL cells normalized to the 18S rRNA levels. **(C) **uPA protein secretion in chondrocytes was determined by sandwich ELISA.

The effects of macrophages on the expression of uPA in human chondrocytes were evaluated under PB-MCM stimulation. Figure 1A shows the dose-dependent induction of uPA transcripts by PB-MCM in human chondrocytes. The time courses determined for the uPA mRNA levels revealed an increase after 30 minutes of PB-MCM stimulation and a peak expression at 2 hours, followed by a gradual reduction thereafter (Figure 1B). The exposure of chondrocytes to PB-MCM caused significant increases in the uPA secretion levels from human chondrocytes (Figure 1C).

### PB-MCM-induced uPA expression is mediated by the JNK and Akt signaling pathways

**Figure 2:**
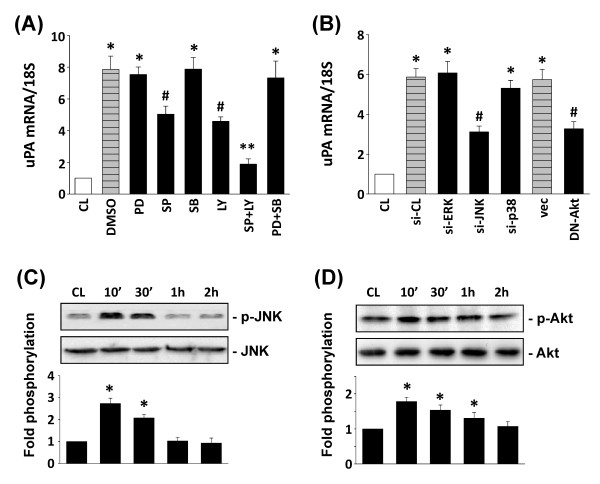
**The JNK and Akt pathways are required for peripheral blood-macrophage-conditioned medium (PB-MCM)-induced urokinase-type PA (uPA) expression**. Cells were untreated (CL) or stimulated with PB-MCM for 2 hours. The results shown are the mean ± SEM from three to four independent experiments. Before culturing as CL or PB-MCM-stimulated cells, chondrocytes were pretreated with PD98059 (PD; 30 μ*M*), SP600125 (SP; 20 μ*M*), SB203580 (SB; 10 μ*M*), or LY294002 (LY, 30 μ*M*) individually or in combination for 1 hour **(A)**, or transfected with control siRNA (si-CL), control pcDNA3 vector (vec), or siRNAs targeting ERK (si-ERK), JNK (si-JNK), p38 (si-p38), or dominant negative mutant of Akt (DN-Akt) **(B)**. *P < 0.05 versus CL. ^#^*P *< 0.05 versus DMSO-treated, control siRNA (si-CL)-, or control pcDNA3 vector (vec)-transfected cells under PB-MCM stimulation. **P < 0.05 versus SP- or LY-pretreated cells under PB-MCM stimulation. **(C, D) **Control (CL) or PB-MCM-stimulated chondrocytes were maintained for the times indicated. The phosphorylation of JNK (C) and Akt (D) in these cells is indicated by the band densities (normalized to total protein levels) relative to CL. *P < 0.05 versus CL cells.

The MAPK superfamily and PI3K/Akt pathways are known to regulate gene expression and specific cellular functions [20,21]. To determine whether PB-MCM-induced uPA expression is mediated through the MAPK- or PI3K/Akt-dependent pathways, human chondrocytes were exposed to specific inhibitors of ERK (PD98059; 30 μ*M*), JNK (SP600125; 20 μ*M*), p38 (SB203580; 10 μ*M*), or PI3K (LY294002, 30 μ*M*) for 1 hour before and during stimulation with PB-MCM. The PB-MCM-induced uPA mRNA expression in chondrocytes (Figure 2A) was significantly inhibited by SP600125 and LY294002, but not by PD98059 and SB203580. Treatment of chondrocytes with a combination of SP600125 and LY294002 resulted in the additive inhibition of PB-MCM-induced uPA expression. To confirm further the involvement of JNK and Akt, but not ERK and p38, in the modulation of uPA expression by PB-MCM stimulation, we examined the effects of expressing specific MAPK siRNAs, and a DN-Akt plasmid on PB-MCM-induced uPA expression in chondrocytes. PB-MCM-induced uPA mRNA expression (Figure 2B) was inhibited by JNK-specific siRNA and DN-Akt, but not by ERK-, p38-, or control siRNAs (100 μmol/ml for each), or the pcDNA3 empty vector. The phosphorylation of JNK (Figure 2C) and Akt (Figure 2D) in chondrocytes increased rapidly after PB-MCM stimulation, reaching maximal levels at 10 minutes. After such transient increases, the levels of phosphorylation decreased to nearly basal levels.

### NF-κB binding sites are major determinants of the PB-MCM-induction of uPA promoter activity

**Figure 3:**
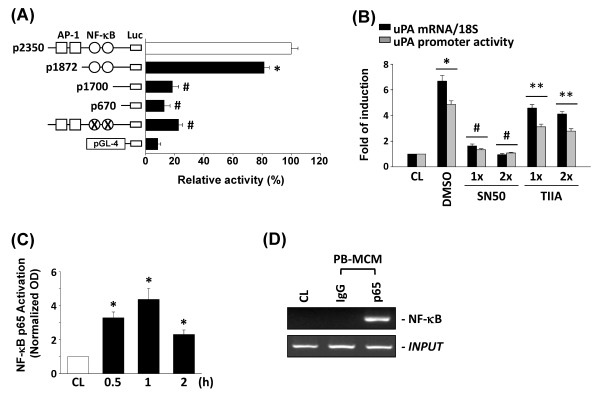
**The role of nuclear factor (NF)-κB in peripheral blood-macrophage-conditioned medium (PB-MCM)-induced urokinase-type PA (uPA) mRNA expression and promoter activity**. **(A) **Left panel, uPA promoter p2350-Luc plasmid, deletion, and mutant promoter constructs. Right panel, PromoCell HCs were cotransfected with uPA promoter constructs and stimulated with PB-MCM for 2 hours. Promoter activities were measured by using a luciferase assay normalized to β-galactosidase activity. They are shown relative to the activities in cells transfected with p2350-Luc (set to 100%). *P < 0.05 versus p2350-Luc. ^#^*P *< 0.01 versus p2350-Luc. **(B) **uPA mRNA and uPA p2350-Luc activity in chondrocytes wase determined in cells pretreated with vehicle (DMSO), different doses of NF-kB inhibitor SN50, or AP-1 inhibitor Tanshinone IIA (TIIA), and then stimulated with PB-MCM for 2 hours. *P < 0.05 versus CL. ^#^*P *< 0.01 versus DMSO control with PB-MCM stimulation. **P < 0.05 versus DMSO control with PB-MCM stimulation. (C) NF-κB p65 activation determined with TF ELISA. All bar graphs are indicative of fold-changes compared with control chondrocytes (CL). *P < 0.05 versus CL. **(D) **ChIP analysis of NF-κB by using a p65 antibody.

The human uPA gene promoter contains multiple transcription factor binding sites, including those for AP-1 and nuclear factor (NF)-κB [22]. To elucidate the *cis*-acting elements in the uPA gene promoter that mediate PB-MCM-induced uPA transcription, luciferase assays were conducted by using the p2350-Luc plasmid and several deletion or mutant promoter constructs (Figure 3A). In human chondrocytes, the -2,350/+30 region of the uPA promoter directed maximal luciferase activity. Sequence deletions from -2,350 to -1,872 (which incorporate AP-1 binding sites) slightly impaired PB-MCM-induced uPA promoter activity (about 20% lower than the p2350-Luc). Further deletions from -1,872 to -1,700 (NF-κB binding sites) and mutations in NF-κB binding sites, however, reduced PB-MCM-induced uPA promoter activity by more than 80% (Figure 3A) compared with p2350-Luc. We further tested whether NF-κB and AP-1 activations are involved in the signal-transduction pathway leading to PB-MCM-induced uPA gene expression. Human chondrocytes were incubated with a specific inhibitor for NF-κB (SN50, 100 and 200 n*M*) or AP-1 (Tanshinone IIA, 3 and 6 μ*M*) for 1 hour, which was followed by stimulation with PB-MCM for 2 hours. The PB-MCM-induced uPA mRNA expression levels and uPA promoter activity in chondrocytes (Figure 3B) was significantly reduced through inhibition with SN50, and partially inhibited with Tanshinone IIA, indicating that NF-κB is the major transcription factor involved in the regulation of uPA gene induction.

To investigate whether NF-κB binds the uPA promoter region in human chondrocytes, we performed quantitative analysis of the NF-κB p65 binding activity *in vitro *by using TF ELISA kits from Panomics. The treatment of chondrocytes with PB-MCM caused increased NF-κB p65-DNA binding activity after 0.5 hours, which remained elevated for at least 1 hour (Figure 3C). These results were confirmed by ChIP analysis. Chromosomal DNA immunoprecipitated with a p65 antibody was subjected to PCR by using primers designed to amplify the uPA promoter region harboring the NF-κB binding site. NF-κB was indeed found to bind to the uPA promoter region containing the NF-κB consensus sites (Figure 3D).

### The JNK and Akt signaling pathways are involved in macrophage-induced uPA promoter activity

**Figure 4:**
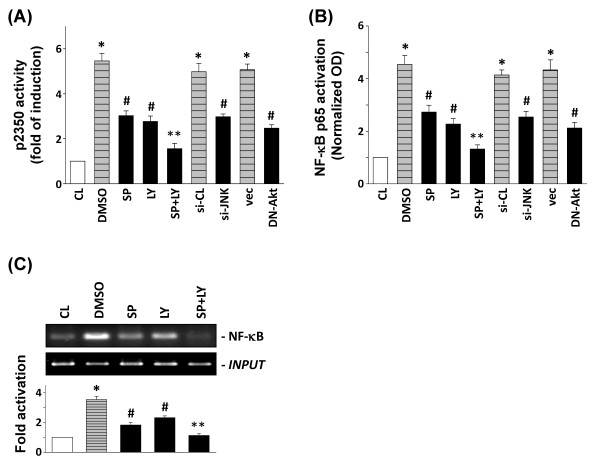
**The JNK and Akt signaling pathways are involved in peripheral blood-macrophage-conditioned medium (PB-MCM)-induced urokinase-type PA (uPA) promoter activity**. Chondrocytes were maintained as controls (CL) or pretreated with vehicle (DMSO), with SP600125 (SP) and LY294002 (LY) individually or in combination for 1 hour, or transfected with control siRNA (si-CL), control pcDNA3 vector (vec), JNK-siRNA (si-JNK), or DN-Akt plasmid, and then stimulated with PB-MCM. **(A) **uPA-2350-Luc activity was determined with luciferase assay after stimulation with PB-MCM for 2 hours. **(B) **NF-κB p65 activation was determined by TF ELISA after stimulation with PB-MCM for 1 hour. **(C) **NF-κB p65 binding to uPA promoter in chondrocytes after 1 hour PB-MCM stimulation was analyzed with ChIP assay. Cells were pretreated with vehicle (DMSO), or with SP600125 (SP) and LY294002 (LY) individually or in combination for 1 hour. The ChIP assay was performed by using p65 antibody. All bar graphs indicate the fold changes compared with CL chondrocytes, mean ± SEM. *P < 0.05 versus CL. ^#^*P *< 0.05 versus DMSO control, si-CL, or vec-transfected cells with PB-MCM stimulation. **P < 0.05 versus SP- or LY-pretreated cells under PB-MCM stimulation.

To evaluate whether the inhibition of uPA expression by the JNK and Akt signaling pathways occurs at the transcriptional level, we studied the effects of specific inhibitors, siRNA molecules that target JNK, and a DN-Akt on PB-MCM-induced uPA p2350-Luc promoter (Figure 4A) and NF-κB p65 (Figure 4B) activities. Culturing of the chondrocytes in PB-MCM increased the p2350-Luc and NF-κB p65 activities by 5.5- and 4.5-fold, respectively, compared with unstimulated cells and after normalization with a transfection control. Pretreatment of the cells with SP600125 and LY294002, or transfection with JNK-siRNA and DN-Akt, resulted in a marked inhibition of both the PB-MCM-induced uPA promoter activity (Figure 4A) and NF-κB p65 activation (Figure 4B). Pretreatment with SP600125 and LY294002 caused a simultaneous and additive inhibition of PB-MCM-induced p2350-Luc and NF-κB p65 activities.

ChIP analysis further revealed that the pretreatment of human chondrocytes with SP600125 and LY294002 inhibits the PB-MCM induction of NK-κB p65 promoter binding activity (Figure 4C). The combined treatment of chondrocytes with SP600125 and LY294002 resulted in the additive inhibition of PB-MCM-induced p65 promoter binding activity (Figure 4C).

### IL-1ra inhibits macrophage-induced signaling transduction and uPA expression

**Figure 5:**
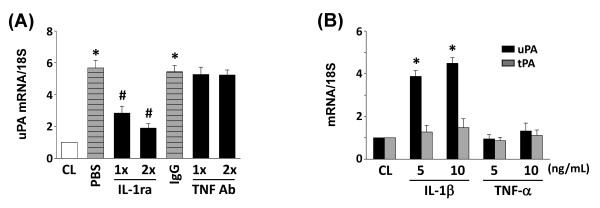
**Interleukin (IL)-1β is the major factor underlying peripheral blood-macrophage-conditioned medium (PB-MCM)-induced urokinase-type PA **(**uPA) expression in chondrocytes**. RNA samples were then isolated and subjected to real-time PCR analysis. The mRNA data are presented as the fold changes in fluorescent intensities compared with control (CL) chondrocytes normalized to the 18S rRNA levels. **(A) **Chondrocytes were untreated (CL) or stimulated with PB-MCM for 2 hours. Before culturing under control conditions (CL) or stimulation with PB-MCM, the PB-MCM and chondrocytes were preincubated with PBS, IL-1ra, isotype-matched IgG, or neutralizing antibody against TNF-α (TNF Ab) individually for 2 hours. *P < 0.05 versus CL. ^#^*P *< 0.05 versus PBS, IgG-, or TNF Ab-treated cells under PB-MCM stimulation. **(B) **Chondrocytes cultured as unstimulated CL, or stimulated with different doses of IL-1b or TNF-a for 2 hours. *P < 0.01 versus CL.

**Figure 6:**
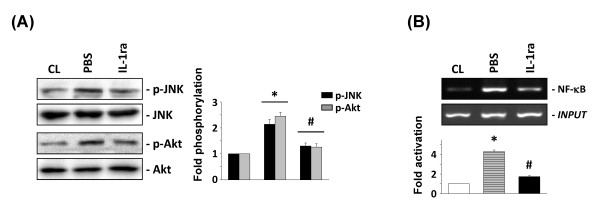
**Effects of interleukin (IL)-1ra on peripheral blood-macrophage-conditioned medium (PB-MCM)-induced signaling in chondrocytes**. Chondrocytes were untreated (CL) or stimulated with PB-MCM for the indicated periods (*t*). The results are the mean ± SEM of three to four separate experiments and show the effects of IL-1ra on PB-MCM-induced JNK and Akt phosphorylation (**A**, *t *= 10 min) and NF-κB activation (**B**, *t *= 1 hour), respectively. Before culturing under control conditions (CL) or stimulation with PB-MCM, the PB-MCM and chondrocytes were preincubated with 1 × PBS or IL-1ra for 2 hours. *P < 0.05 versus CL. ^#^*P *< 0.05 versus PBS-treated cells under PB-MCM stimulation.

IL-1β and TNF-α are major secreted products of macrophages [3]. The incubation of human chondrocytes with IL-1-receptor antagonists (IL-1ra, 1 and 2 μg/ml), but not TNF-α neutralizing antibody (20 and 40 μg/ml), significantly inhibited PB-MCM-induced uPA mRNA expression (Figure 5A). Human chondrocytes directly stimulated with TNF-α had minor effects on the expression of uPA and tPA (Figure 5B). However, stimulation of chondrocytes with IL-1β had similar effects on uPA expression to PB-MCM (Figure 5B). The phosphorylation of JNK and Akt was simultaneously eliminated by pretreating the human chondrocytes with IL-1ra (Figure 6A), which also inhibited PB-MCM-induced NF-κB promoter binding activity (Figure 6B).

### Exposure of human chondrocytes to shear stress of 2 and 5 dyn/cm^2 ^inhibits macrophage-induced uPA expression

**Figure 7:**
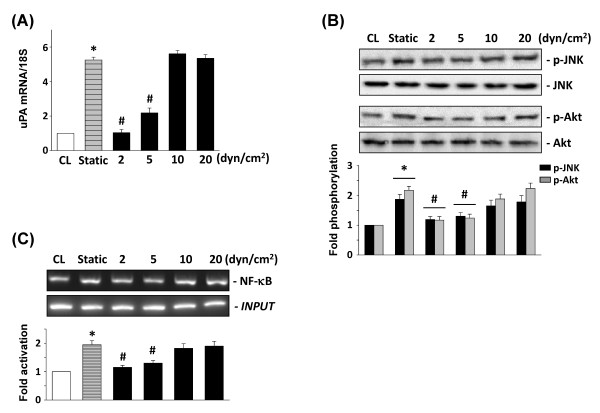
**Exposure of chondrocytes to lower shear stress inhibits peripheral blood-macrophage-conditioned medium (PB-MCM)-induced urokinase-type PA (uPA) expression. **Chondrocytes were kept as controls (CL), or subjected to different levels of shear stress with PB-MCM stimulation. Static chondrocytes were stimulated with PB-MCM without shearing (static). All bar graphs represent fold differences compared with control chondrocytes (CL), mean ± SEM. *P < 0.05 versus CL cells. #P < 0.05 versus static chondrocytes with PB-MCM stimulation. (A) uPA mRNA levels determined by real-time PCR analysis. The mRNA data are presented as the fold changes in fluorescent intensities compared with CL cells normalized to the 18S rRNA levels. (B) The phosphorylation of JNK and Akt after 10 minutes of PB-MCM stimulation was determined by Western blotting. The results are mean ± SEM. All bar graphs represent folds of CL and are normalized to total JNK or Akt. (C) NF-κB p65 binding to the uPA promoter after 1 hour of PB-MCM stimulation was analyzed by ChIP assay by using a p65 antibody. All bar graphs represent folds of CL and are normalized based on the input DNA.

Stimulation of human chondrocytes with PB-MCM under static conditions increases uPA expression (Figure 7A). Exposure of chondrocytes cultured in PB-MCM to shear stress at 2 and 5 dyn/cm^2 ^was found to significantly inhibit PB-MCM-induced uPA mRNA expression. However, shear stresses at higher levels of 10 and 20 dyn/cm^2 ^did not have such inhibitory effects (Figure 7A). Exposure of chondrocytes to shear stresses of 2 and 5 dyn/cm^2^, but not 10 and 20 dyn/cm^2^, resulted in a marked inhibition of the PB-MCM-induced JNK and Akt phosphorylation (Figure 7B), and also of p65 NF-κB-DNA binding activity (Figure 7C).

### Effect of AMPK on PB-MCM-induced uPA expression

**Figure 8:**
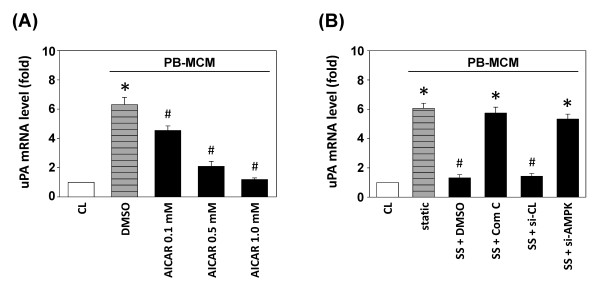
**Effect of AMPK on peripheral blood-macrophage-conditioned medium (PB-MCM)-induced urokinase-type PA **(**uPA) expression**. All bar graphs represent folds of control chondrocytes (CL) and are normalized to 18S rRNA. The results are shown as mean ± SEM. **(A) **Chondrocytes were kept as CL or stimulated with PB-MCM for 2 hours. Before being kept as CL or stimulated with PB-MCM, cells were pretreated with 0.5 to 1 m*M *AICAR for 2 hours. *P < 0.05 versus CL chondrocytes. ^#^*P *< 0.05 versus DMSO-treated chondrocytes (vehicle control) with PB-MCM stimulation. **(B) **Chondrocytes were kept as CL, or subjected to 2 dyn/cm^2 ^of shear stress (SS) with PB-MCM stimulation. Cells were treated with 10 m*M *compound C or transfected with si-CL or si-AMPK. Static chondrocytes were stimulated with PB-MCM without shearing (static). *P < 0.05 versus CL, vesicle control (SS + DMSO), or control siRNA-transfected (SS + si-CL) cells with PB-MCM stimulation. ^#^*P *< 0.05 versus compound C-treated (SS + com C) or AMPK siRNA-transfected (SS + si-AMPK) cells with PB-MCM stimulation.

A recent study showed that AMPK plays an important role in regulating cell function and inflammation in chondrocytes [23]. We investigated whether the PB-MCM-induced uPA expression is modulated by AMPK. Chondrocytes were incubated with different doses of AMPK activator AICAR for 2 hours before and during stimulation with PB-MCM. The PB-MCM-induced mRNA expression of chondrocyte uPA was significantly inhibited by 0.5 to 1 m*M *AICAR treatment (Figure 8A). Conversely, the addition of 10 m*M *compound C or the transfection of AMPK siRNA before exposure to shear stress at 2 dyn/cm^2 ^abolished the shear-mediated inhibition of uPA expression (Figure 8B). These results indicated that AMPK plays an important role in the PB-MCM-induction and shear-inhibition of uPA expression in chondrocytes.

## Discussion

Increasing evidence suggests that catabolic genes in chondrocytes play an important role in the onset of OA in cartilage [1]. Previous studies also demonstrated a pivotal role for shear stress in regulating gene expression and function in chondrocytes [14,16,24-26]. The novel findings of our present study are as follows:

1. Conditioned medium from peripheral blood macrophages increases uPA expression in human chondrocytes;

2. This increase in uPA expression is particularly attributable to the paracrine effects of the cytokine IL-1β released by macrophages;

3. Macrophage-induced uPA expression in chondrocytes is mediated through JNK and Akt phosphorylation, and NF-κB activation; and

4. Lower shear stresses attenuate peripheral blood macrophage-induced uPA expression.

uPA is a serine protease that converts plasminogen to plasmin. Plasmin can then degrade proteoglycans and transform MMPs into their active forms. The uPA itself also has a direct role in the degradation of ECM proteins [7]. The PA/plasmin system has a broad spectrum of activity. In human OA and animal models of OA, where enhanced bone remodeling may trigger cartilage damage, uPA/plasmin is upregulated [27]. Other reports also indicated a higher expression and activity of uPA in arthritis groups compared with normal controls [28,29]. The increased levels of uPA in OA joints suggest that they play a role in this disease. It has been demonstrated that the transcript levels of uPA increase significantly during the early and medium stages of OA [30]. The ability of macrophages to stimulate uPA gene expression in chondrocytes may, at least in part, lead to the elevation of uPA in the synovial fluid during OA progression. The mechanism by which macrophages regulate uPA gene expression in chondrocytes, however, remains unclear.

In our present study, we investigated the molecular mechanisms by which macrophages stimulate uPA expression in human chondrocytes. We provide several lines of evidence from our current data that macrophage-induced uPA expression in chondrocytes is mediated via NF-κB. First, we found that PB-MCM stimulates uPA expression and production by human chondrocytes in an *in vitro *culture system. Second, TF ELISA and ChIP assays demonstrated an increase in NF-κB binding to the uPA gene promoter in chondrocytes. Third, the inhibition of NF-κB activation in chondrocytes by pretreatment with JNK and Akt inhibitors, transfection with specific siRNAs of JNK, or the expression of a dominant-negative mutant of Akt, abolishes macrophage-induced uPA expression.

The results of our present study also demonstrate for the first time that macrophages not only promote the secretion of uPA, but also induce their gene expression in cultured human chondrocytes, and that macrophage-induced uPA expression occurs at the transcriptional level. Analysis of human uPA promoter activity with different plasmid constructs further revealed that NF-κB is the major *cis *element for PB-MCM responsiveness via JNK and Akt phosphorylation. It was reported previously that the expression of inflammatory genes in chondrocytes is controlled by different signaling pathways, which leads to activation of the MAPKs and PI3K, and of the transcriptional regulator NF-κB [31,32]. Based on our current results, we propose a possible mechanism by which macrophages induce JNK and Akt phosphorylation in chondrocytes, which in turn promotes NF-κB binding to the uPA promoter and its subsequent transcriptional activation.

Cartilage destruction in arthritis is somehow affected by interactions between chondrocytes and macrophages. Chondrocytes increase their secretion of catabolic enzyme activities after exposure to macrophages [2], whereas the activation of MMP-9 produced by macrophages is dependent on chondrocyte-derived factors [33]. It has been shown that synovial tissue from early OA patients contains more macrophages, which may suggest that inflammation is at higher levels during the early phases of OA [34]. Macrophage derived-cytokines might therefore play a crucial role in the onset and progression of OA. IL-1β and TNF-α are associated with the development of early arthritis, whereas IL-1β maintains the inflammatory reaction in later stages [35]. It has been suggested that, in the osteoarthritis synovium, both inflammatory and destructive responses are dependent largely on macrophages and that these effects are cytokine-driven through a combination of IL-1 and TNF-α [36]. IL-1β has also been reported to have synergistic effects with other cytokines that regulate catabolic gene expression in human chondrocytes [37,38]. IL-1β has been considered the central mediator of cartilage loss in OA by upregulating the extracellular proteolytic enzymes in cartilage degradation, such as MMPs and aggrecanases [39]. Additionally, it has been reported that uPAR, which is involved in cartilage degradation by serine proteinases and is upregulated in OA, is stimulated on chondrocytes in a dose-dependent manner by IL-1β [40]. Although the effect of IL-1b on chondrocyte has been extensively studied, and inflammatory macrophages and the mediators they release have been implicated in the pathology of OA, the detailed mechanism of macrophage-induced uPA expression in human chondrocytes remains unclear. The increases in uPA expression in chondrocytes induced by PB-MCM suggest that macrophages may release soluble mediators to exert paracrine effects on chondrocytes and thereby induce uPA expression.

Our current data further indicate that TNF-α is not a major mediator of uPA expression in chondrocytes. The inhibitory effects of IL-1ra on the PB-MCM-induced activation of NF-κB and uPA expression in chondrocytes suggest that the effects of PB-MCM are mediated by the binding of IL-1β to their cognate receptors in these cells. Our results propose a possible signal-transduction pathway in chondrocytes in which macrophages release IL-1b, which induces JNK and Akt phosphorylation, and NF-kB activation, thus resulting in uPA transcriptional activation, expression, and secretion. Previous studies have shown that IL-1b is responsible for the inflammatory gene upregulation in human chondrocytes through activation of the JNK or Akt, and NF-kB signaling pathways [41,42]. Here we showed that IL-1b is the critical factor in PB-MCM-induced uPA expression of chondrocytes through activation of JNK and Akt simultaneously. The present study demonstrated that AP-1 is also involved, but plays lesser roles, in PB-MCM-induced uPA expression (Figure 3). Therefore, our results suggest that IL-1β-induced signaling may be a major factor in PB-MCM-induced uPA expression and that other signaling pathways induced by macrophages may also have minor roles in regulating uPA expression in chondrocytes.

Mechanical stimulation is well recognized as having regulatory effects on different cell types, including tumor cells [17], chondrocytes [14], and vascular cells [19] derived from tissues normally exposed to mechanical forces. It has been reported that physiologicl levels of shear stress play important roles in vascular endothelial function and gene expression. Higher levels of shear stress (>15 dyne/cm^2^) induce endothelial quiescence and atheroprotective gene expression, whereas lower shear stress (<4 dyne/cm^2^) stimulates an atherogenic phenotype [43,44]. Previous studies also demonstrated that high shear stress (20 dyn/cm^2^) significantly inhibits proinflammatory factor- or smooth muscle cell-induced expression of inflammatory genes in endothelial cells [19,45]. The pressure that is applied to joints comprises a complex combination of strain, shear stress, and compressive forces [46]. However, whereas compressive or hydrostatic forces have been studied and shown to be beneficial at specific frequencies and levels, the effects of shear stress on chondrocytes remain controversial. In addition, exercise has been shown to improve pain and function in OA and is recommended by the Osteoarthritis Research Society International (OARSI) for the management of hip and knee OA [47]. However, to date, very little research has been conducted to investigate whether physiological shear stress can also be used to prevent the onset of OA. Fluid shear stress has been shown to activate proinflammatory genes such as cyclooxygenase-2, prostaglandins (PGs), and IL-6 [24,32]. It has been demonstrated that exposure of human chondrocytes to high shear stress, Toll-like receptor 4 (TLR4), and caveolin-1 is upregulated by sequential expression of microsomal PGE synthase-1 and L-PGD synthase. TLR4 and caveolin-1 exert antagonistic effects on IL-6 synthesis; and further regulate the activity of ERK1/2, PI3K, protein kinase A, and NF-κB-dependent IL-6 expression in sheared chondrocytes [48].

In addition, another study has reported that high fluid shear stress induces IL-1β and 15-deoxy-Δ12,14-prostaglandin J2 synthesis, which antagonistically regulate MMP-9 expression via PI3K-, ERK1/2-, -PPARγ-, and JNK-dependent NF-κB-activating pathways in human chondrocytes at short and long shear-exposure times [49]. Previous studies have largely focused on shear stress-induced NF-κB activation and the following effects on chondrocyte catabolic gene expression, but the modulating effect of shear stress on inflammatory stimuli-induced uPA expression in chondrocytes has not been elucidated.

Our present study reveals for the first time that macrophage-induced uPA upregulation is inhibited in chondrocytes subjected to lower levels of shear stress. Exposure of chondrocytes to shear stress of 2 dyn/cm^2^, but not 20 dyn/cm^2^, significantly inhibits peripheral blood macrophage-induced signal transduction and uPA expression. Hence, lower shear stress may exert chondroprotective effects by suppressing macrophage-induced uPA expression.

AMPK has been recognized to exert antiinflammatory effects in multiple tissues [50]. It is activated in response to physiological stimuli such as nutrient deprivation, hypoxia, or shear stress [51,52]. Some evidence supports a role of AMPK in the regulating of cell function in chondrocytes. AMPK activators inhibited IL-1β- and TNF-α-induced expression of procatabolic genes in chondrocytes [23]. In addition, inhibition of AMPK increases chondrocyte sensitivity to the induction of apoptosis [53]. In this study, administration of AICAR significantly suppressed PB-MCM-induced uPA expression. Exposure of chondrocytes to shear stress at 2 dyn/cm^2 ^inhibited PB-MCM-induced uPA expression, and this shear effect was blocked by treatment of compound C and transfection of AMPK siRNA. Thus, our findings indicate that AMPK activity may contribute to shear-mediated antiinflammatory effects in human chondrocytes.

## Conclusions

In summary, we here demonstrate for the first time that different levels of shear stress have regulatory effects on inflammatory stimuli-induced gene expression in chondrocytes. Our analyses have also identified a unique molecular mechanism of macrophage-induced JNK and Akt phosphorylation, NF-κB activation, and uPA expression in chondrocytes. These findings provide a molecular basis for the mechanisms underlying the protective function of shear stress against uPA induction. Because macrophage infiltration and uPA upregulation are principal features of early-stage OA, our current data have potential relevance for cartilage tissue engineering and future therapeutic interventions in arthritis patients.

## Abbreviations

AMPK: AMP-activated protein kinase; ChIP: chromatin immunoprecipitation assay; ECM: extracellular matrix; ELISA: enzyme-linked immunosorbent assay; IL-1β: interleukin-1β; MACS: magnetic-activated cell sorting; MMP: matrix metalloproteinase; NF-κB: nuclear factor-κB; OA: osteoarthritis; PA: plasminogen activator; PBMC: peripheral blood mononuclear cell; PB-MCM: peripheral blood-macrophage conditioned medium; TNF-α: tumor necrosis factor-α; uPA: urokinase plasminogen activator.

## Competing interests

The authors declare that they have no competing interests.

## Author's contributions

CCY and CNC conceived the study, had full access to all the data, and wrote the manuscript. SFC, TYH, HIC, HCK, and YCW performed most of the experiments and acquired the data. CHH and CSS contributed new reagents or analytic tools. All authors read and approved the final manuscript.
